# The involvement of 4‐1BB/4‐1BBL signaling in glial cell‐mediated hypothalamic inflammation in obesity

**DOI:** 10.1002/2211-5463.12426

**Published:** 2018-04-19

**Authors:** Jiye Kim, Yoon‐Hee Kwon, Chu‐Sook Kim, Thai H. Tu, Byung‐Sam Kim, Yeonsoo Joe, Hun T. Chung, Tsuyoshi Goto, Teruo Kawada, Taesun Park, Myung‐Sook Choi, Min‐Seon Kim, Rina Yu

**Affiliations:** ^1^ Department of Food Science and Nutrition University of Ulsan South Korea; ^2^ Department of Biological Science University of Ulsan South Korea; ^3^ Graduate School of Agriculture Kyoto University Uji Japan; ^4^ Department of Food and Nutrition Yonsei University Seoul South Korea; ^5^ Department of Food Science and Nutrition Center for Food and Nutritional Genomics Research Kyungpook National University Daegu South Korea; ^6^ Division of Endocrinology and Metabolism University of Ulsan College of Medicine Seoul South Korea

**Keywords:** hypothalamus, inflammation, obesity

## Abstract

Obesity‐induced inflammation occurs not only in peripheral tissues but also in areas of the central nervous system. Glial cells such as astrocytes and microglia play crucial roles in obesity‐related hypothalamic inflammation, leading to the derangement of energy metabolism and neurodegenerative pathologies. Here, we show that the interaction of 4‐1BB/4‐1BBL between lipid‐laden astrocytes/microglia promotes hypothalamic inflammation in obesity. Stimulation of 4‐1BB, a member of the TNF receptor superfamily, and/or its ligand 4‐1BBL on astrocytes and/or microglia with a specific agonist resulted in activation of the inflammatory signaling pathway and enhanced production of inflammatory mediators. Contact coculture of lipid‐laden astrocytes and microglia increased the production of inflammatory mediators, and blockade of the 4‐1BB/4‐1BBL interaction reduced the inflammatory response. Moreover, deficiency of 4‐1BB reduced hypothalamic inflammation in obese mice fed an high‐fat diet. These findings suggest that 4‐1BBL/4‐1BB signaling enhances the glial cell‐mediated inflammatory cross talk and participates in obesity‐induced hypothalamic inflammation.

**Abbreviations**
ATCM: adipose tissue‐conditioned medium
C‐As: control‐astrocyte
FFA: free fatty acid
HFD: high‐fat diet
LL‐As: lipid‐laden astrocyte
MS: multiple sclerosis
TNFRSF: TNF receptor superfamily

Obesity‐induced inflammation occurs not only in peripheral tissues (e.g., adipose tissue, liver, pancreas, and skeletal muscle) but also in areas of the central nervous system (CNS) such as the hypothalamus 1. A growing body of evidence shows that obesity and/or high‐fat diet (HFD) feeding increases transcription of inflammatory cytokines in the hypothalamus accompanied by accumulation and activation of glial cells, a process referred to as gliosis 2, 3. The increased inflammatory microenvironment disturbs leptin and/or insulin signaling and enhances neurotoxicity in the hypothalamus, and hence, obesity‐induced hypothalamic inflammation is implicated in the dysregulation of energy homeostasis, leptin/insulin resistance, and neurodegenerative pathologies 4. Understanding the molecules and cellular mechanisms involved in obesity‐induced hypothalamic inflammation is essential to develop new therapeutic targets against metabolic pathologies.

The hypothalamus contains neurons and a range of resident non‐neuronal glial cells including microglia and astrocytes. Microglia play an important role as resident immunocompetent phagocytic cells in various inflammatory CNS pathologies 5, 6, whereas astrocytes, which are the most abundant cells in the hypothalamus, provide a nurturing environment regulating all aspects of neuronal function, including synaptic plasticity, survival, development, and glucose/lipid metabolism 7, 8. Both microglia and astrocytes play important roles in various inflammatory pathological processes by increasing their reactivity and causing neuronal damage, and glial cell–cell interactions are also implicated in the induction and maintenance of hypothalamic inflammation 9. Interestingly, HFD feeding and obesity increase inflammatory reactivity of both glial cells and the expression of inflammatory cytokines 10, 11, and restraining inflammatory activation by depleting microglia or by depleting inflammatory signaling attenuates obesity phenotypes in mice 12. Moreover, we recently demonstrated that astrocytes accumulate lipid droplets under free fatty acid (FFA)‐rich environments such as in the obese condition, and the lipid‐laden astrocytes‐derived inflammatory mediators enhance microglia migration and activation 13. However, the potential cross talk between astrocytes and microglia in obesity‐induced hypothalamic inflammation has not yet been explored.

4‐1BB (also known as CD137) is a costimulatory and inflammatory receptor that is expressed on activated T cells 14 and some of nonimmune cells such as endothelial cells and adipocytes 15. 4‐1BB ligand (4‐1BBL, also known as CD137L) is highly expressed on macrophages and antigen‐presenting cells and can receive and transmit reverse signals into cells by binding to its receptor, 4‐1BB 16, 17. 4‐1BBL‐deficient mice were shown to exhibit profoundly less microglia activation during experimental autoimmune encephalomyelitis 18, which is a well‐established murine model for neuroinflammation and human multiple sclerosis (MS). Hence, it is conceivable that 4‐1BB/4‐1BBL may participate in glial cell‐mediated hypothalamic inflammation. Of note, our previous study demonstrated that 4‐1BB and/or 4‐1BBL enhance obesity‐induced peripheral inflammation such as adipose tissue (adipocytes/macrophages) and skeletal muscle (myotubes/macrophages) by providing a bidirectional inflammatory signal 17, 19. However, the association of these molecules with obesity‐induced hypothalamic inflammation remains completely unknown. As astrocytes express 4‐1BB and microglia express 4‐1BBL 18, 20, we hypothesized that these molecules may have the potential to modulate cross talk between astrocytes and microglia and thus contribute to obesity‐induced hypothalamic inflammation.

In this study, we demonstrated that 4‐1BB/4‐1BBL signaling increases inflammatory reactivity of astrocytes and microglia to produce inflammatory mediators, leading to hypothalamic inflammation in obese condition. 4‐1BB/4‐1BBL signaling may be a potential target to protect against obesity‐related hypothalamic inflammation and metabolic/neurodegenerative diseases.

## Materials and methods

### Animals

Nine‐week‐old male 4‐1BB‐deficient mice on a C57BL/6 background were establish at the Immunomodulation Research Center of University of Ulsan, South Korea 21. 4‐1BB‐deficient mice and their wild‐type (WT) counterparts were fed a HFD (60% of calories from fat; Research Diets Inc., New Brunswick, NJ, USA) or a low‐fat diet (10% of calories from fat; Research Diets Inc.) for 8 weeks and given free access to food and water. The animals were killed by CO_2_ asphyxiation, and the hypothalamus was dissected. All animal experiments were approved by the animal ethics committee of the University of Ulsan (LNY‐16‐010) following National Institutes of Health guidelines.

### Antibodies

Agonistic monoclonal antibody (Ab) against 4‐1BB (3E1) was generated from nude mice that were injected intraperitoneally with a subcloned hybridoma to induce ascites formation 22. The Ab was purified from ascites fluid by affinity column chromatography with protein G Sepharose (Sigma‐Aldrich, St. Louis, MO, USA). Recombinant 4‐1BB Fc (r4‐1BB FC) was purchased from Adipogen (Seoul, Korea). Antagonistic monoclonal Ab against 4‐1BBL (TKS‐1) was purchased from e‐Bioscience (San Diego, CA, USA). Rat immunoglobulin G (Rat IgG) and human IgG1 were purchased from Sigma‐Aldrich and were used as the control.

### Cell cultures and treatments

The microglia cell line BV2 was obtained from the Meta‐Inflammation Basic Research Laboratory (University of Ulsan, Ulsan, Korea). This cell line was maintained in RPMI1640 (Gibco, Grand Island, NY, USA) containing 10% (vol/vol) FBS (Gibco) and incubated at 37 °C in humidified 5% CO_2_. Primary astrocytes were prepared from whole‐brain astrocytes of newborn C57BL/6 mice 23. In brief, brains were ground in the plate in Dulbecco's minimum essential medium supplemented with 10% fetal bovine serum. Cells were collected by centrifugation at 200 ***g*** for 5 min and incubated at 37 °C. Astrocyte purity, as determined by glial fibrillary acidic protein (GFAP) immunohistochemical staining, was > 94%. BV2 (5 × 10^5^ cells/well) or primary astrocytes (4 × 10^5^ cells/well) were treated with obesity‐related factors such as 100 ng·mL^−1^ lipopolysaccharide (LPS), 35 mm glucose, or obese adipose tissue‐conditioned medium (ATCM) in serum‐free RPMI1640 for 6 h, respectively 19. To immobilize r4‐1BB Fc or human IgG1 on culture plates, r4‐1BB Fc or human IgG1 was incubated at 37 °C for 1 h in a CO_2_ incubator, and the wells were rinsed with PBS. The plates were then incubated with RPMI (10% FBS) at 37 °C for 1 h in a CO_2_ incubator, and the well was rinsed with PBS. Microglial cells (BV2) were incubated at 5 × 10^5^ cells/well in wells precoated with 1 μg·mL^−1^ r4‐1BB Fc or human IgG1 for 6 h 17. To stimulate 4‐1BB on primary astrocyte, the cells were incubated with agonistic 4‐1BB Ab (3E1, 1 μg·mL^−1^) or rat IgG for 24 h in serum‐free medium.

### Measurement of cytokine levels

Cytokine levels in culture supernatants were measured using enzyme‐linked immunosorbent assays (ELISA), using a mouse MCP‐1 set (BD Bioscience Pharmingen, San Diego, CA, USA) and a mouse IL‐6 set (R&D Systems, Minneapolis, MN, USA). Values for cytokine levels were calculated from standard curves using the curve‐fitting program SOFTmax (Molecular Devices, Sunnyvale, CA, USA) 17.

### Quantitative real‐time PCR (qRT‐PCR)

Total RNA extracted from cultured cells was reverse transcribed to generate cDNA using M‐MLV reverse transcriptase (Promega, Madison, WI, USA). Real‐time PCR amplification of the cDNA was performed in triplicate with a SYBR premix Ex Taq kit (TaKaRa Bio Inc., Foster, CA, USA) using a Thermal Cycler Dice (TaKaRa Bio Inc., Otsu, Japan). All reactions were performed using the same procedure: initial denaturation at 95 °C for 10 s, followed by 40 cycles of 95 °C for 5 s and 60 °C for 30 s. The relative mRNA levels in each samples were normalized to internal control β‐actin, and calculated by the comparative cycle threshold (Ct) method. Data were analyzed using Thermal Cycler Dice Real Time System Software (Takara Bio, Inc.). The primers used in the analysis are listed in Table 1.

**Table 1:** Mouse primers used in qRT‐PCR analysis

Gene	Forward primer (5′→3′)	Reverse primer (5′→3′)
4‐1BB	CTCTGTGCTCAAATGGATCAGGAA	TGTGGACATCGGCAGCTACAA
4‐1BBL	CCTGTGTTCGCCAAGCTACTG	CGGGACTGTCTACCACCAACTC
MCP‐1	GCATCCACGTGTTGGCTCA	CTCCAGCCTACTCATTGGGATCA
TNFα	AAGCCTGTAGCCCACGTCGTA	GGCACCACTAGTTGGTTGTCTTTG
IL‐6	CCACTTCACAAGTCGGAGGCTTA	GCAAGTGCATCATCGTTGTTCATAC
IL‐1β	TCCAGGATGAGGACATGAGCAC	GAACGTCACACACCAGCAGGTTA
IL‐10	GCCAGAGCCACATGCTCCTA	GATAAGGCTTGGCAACCCAAGTAA
Nos2	CAAGCTGAACTTGAGCGAGGA	TTTACTCAGTGCCAGAAGCTGGA
CD11b	CCACTCATTGTGGGCAGCTC	GGGCAGCTTCATTCATCATGTC
Iba‐1	TGGTCCCCCAGCCAAGA	CCCACCGTGTGACATCCA
GFAP	AGCTAGCCCTGGACATCGAGA	GGTGAGCCTGTATTGGGACAAC
β‐Actin	CATCCGTAAAGACCTCTATGCCAAC	ATGGAGCCACCGATCCACA

### Western blot analysis

BV2 cells were plated at 1.5 × 10^6^ cells/well in 6‐well plates coated with r4‐1BB Fc or human IgG for 30 min. The r4‐1BB Fc‐treated BV2 cells were rinsed with PBS, suspended by scraping in lysis buffer (10 mm Tris/HCl, 10 mm NaCl, 0.1 mm EDTA, 50 mm NaF, 10 mm Na_4_P_2_O_7_, 1 mm MgCl_2_, 0.5% deoxycholate, 1% IGEPAL, protease inhibitors, phosphatase inhibitor cocktail), and centrifuged at 800 ***g*** for 5 min. Samples containing 15–30 μg of total protein were subjected to western blot analysis using polyclonal antibodies to IκB‐α (inhibitor of nuclear factor‐κB alpha; Santa Cruz Biotechnology, Santa Cruz, CA, USA), p‐STAT3 (phospho‐signal transducer and activator of transcription 3; Cell Signaling, Danvers, MA, USA) and β‐actin (Sigma, St. Louis, MO, USA), p‐ERK (p‐extracellular signal‐regulated kinases), total ERK, p‐JNK (c‐jun amino‐terminal kinase), and total JNK (Cell Signaling).

### Statistical analysis

Results are presented as means ± SEM (standard error of the mean). Statistical analyses were performed with GraphPad Prism 5 (San Diego, CA, USA), using Student's *t*‐test or one‐way ANOVA followed by Newman–Keuls test. Differences were considered to be significant at *P* < 0.05.

## Results

### Ablation of 4‐1BB reduces hypothalamic inflammation in obese mice

**Figure 1:**
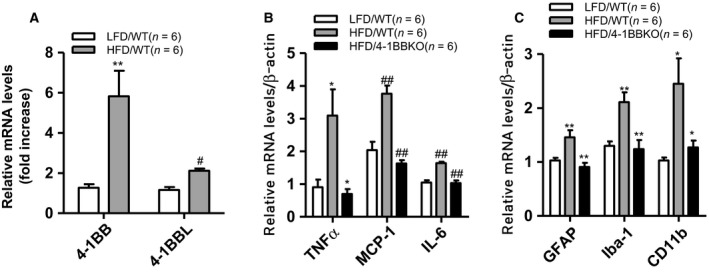
Effects of 4‐1BB deficiency on obesity/HFD‐induced hypothalamic inflammation. WT and 4‐1BBKO mice were fed a HFD for 8 weeks. The transcription levels of (A) 4‐1BB and 4‐1BBL, (B) inflammatory cytokines (TNFα, MCP‐1, IL‐6), and (C) astrogliosis markers (GFAP, Iba‐1, CD11b) were measured by qRT‐PCR. Data are presented as mean ± SEM for *n* = 6. *P < 0.05; **P < 0.01; ^#^ *P* < 0.005; ^##^ *P* < 0.001 compare with low‐fat diet (LFD) or HFD group.

We confirmed that the transcript levels of 4‐1BB and 4‐1BBL significantly increased in the hypothalamus of the HFD‐fed obese mice compared with lean control mice (Fig. 1A). The upregulation of 4‐1BB and 4‐1BBL was accompanied by increased inflammatory markers in the hypothalamus. Levels of inflammatory cytokines (TNFα, MCP‐1, and IL‐6) and activation markers of glial cells significantly increased in hypothalamuses of HFD‐fed obese mice compared with lean controls (Fig. 1B,C). Of note, ablation of 4‐1BB significantly decreased inflammatory markers in the hypothalamus of obese mice fed an HFD; levels of inflammatory cytokines and microglia activation marker (Iba‐1, CD11b) mRNA were significantly lower in the HFD‐fed 4‐1BB‐deficient mice compared with WT obese control (Fig. 1B,C). Microglia activation marker (Iba‐1, CD11b) and astrogliosis marker GFAP were also downregulated in 4‐1BB‐deficient HFD‐fed obese mice compared with the WT obese control (Fig. 1C).

### Effect of 4‐1BB stimulation on the inflammatory responses of astrocytes

**Figure 2:**
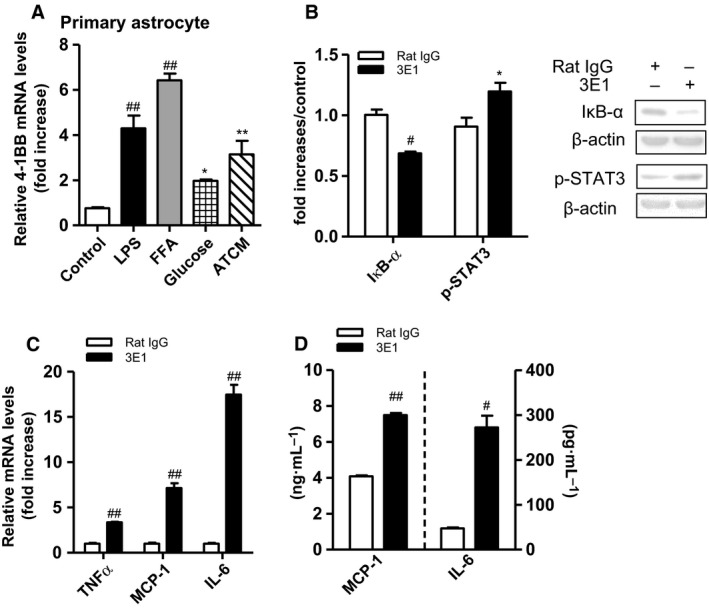
Effect of 4‐1BB stimulation on the inflammatory response of astrocytes. (A) The transcription levels of 4‐1BB. Astrocytes were treated with obesity‐related factors (100 ng·mL^−1^ LPS, 35 mm Glucose, obese ATCM, 200 μm FFA) for 6 h. (B) IκB‐α, p‐STAT3, and β‐actin levels were measured by western blotting. Astrocytes were incubated with agonistic anti‐4‐1BB (3E1, 1 μg mL^−1^) for 20 min. Astrocytes were treated with 1 μg·mL^−1^ 3E1 or rat IgG for 6 and 24 h. (C) Transcription levels of inflammatory cytokines (TNFα, MCP‐1, IL‐6) in 4‐1BB‐stimulated astrocytes were determined by qRT‐PCR. (D) MCP‐1 and IL‐6 proteins were measured by ELISA. Data presented are representative of three independent experiments performed in triplicate. Western blot data shown are the mean ± SEM of three independent experiments performed in duplicate. Representative images of the western blots are shown in the right panel of (B). *P < 0.05; **P < 0.01; ^#^ *P* < 0.005; ^##^ *P* < 0.001 compared with control.

To see the effect of obesity‐related peripheral factors on 4‐1BB expression, primary astrocytes were treated with LPS, FFA, high glucose, and obese ATCM, and the levels of 4‐1BB transcript in the cells were measured by qRT‐PCR. As shown in Fig. 2A, obesity‐related peripheral factors significantly upregulated levels of 4‐1BB transcript in primary astrocytes. To examine whether 4‐1BB on primary astrocytes provided an inflammatory signal, we treated astrocytes with an agonistic 4‐1BB antibody (3E1) for 24 h and then measured the production levels of inflammatory cytokines. The stimulation of 4‐1BB on astrocytes resulted in degradation of IκB‐α and phosphorylation of STAT3, indicating that 4‐1BB signal induced the activation of the intracellular inflammatory signaling pathway in the cells (Fig. 2B). Along with this, we found that 4‐1BB stimulation of astrocytes markedly increased the levels of pro‐inflammatory cytokines transcripts such as TNFα, MCP‐1, and IL‐6 (Fig. 2C), as well as enhancing the release of their protein levels (MCP‐1, IL‐6) (Fig. 2D).

### Effect of 4‐1BBL stimulation on the inflammatory responses of microglia

**Figure 3:**
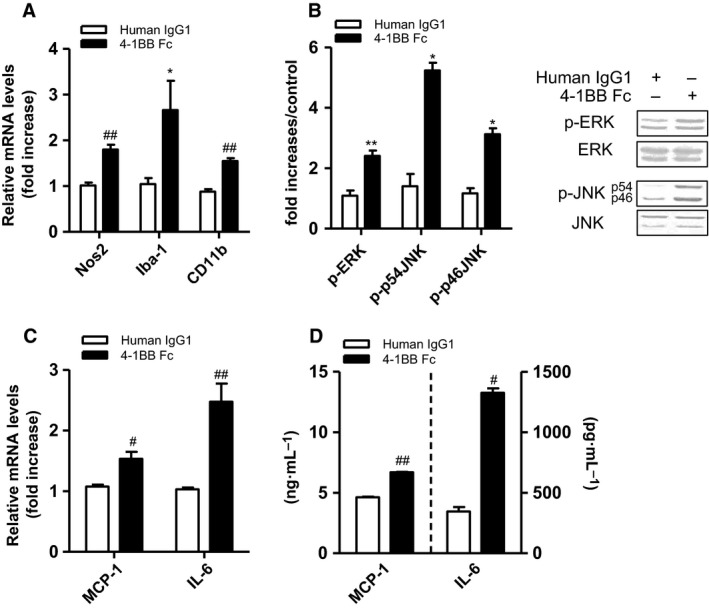
Effects of 4‐1BBL stimulation on the inflammatory response of microglia. Microglia were treated with 1 μg·mL^−1^ r‐4‐1BBFc or human IgG1 (H‐IgG1) for 6 and 24 h. (A, C) The transcription levels of inflammatory cytokines (MCP‐1, IL‐6), Nos2, and microglia activation marker (Iba‐1, CD11b) were determined by qRT‐PCR. (B) p‐ERK, p‐JNK, and β‐actin levels were measured by western blotting. Microglia were incubated with r‐4‐1BBFc (1 μg mL^−1^) for 30 min. (D) MCP‐1 and IL‐6 proteins were measured by ELISA. Data presented are representative of three independent experiments performed in triplicate. Western blot data shown are the mean ± SEM of three independent experiments performed in duplicate. Representative images of the western blots are shown in the right panel of (B). *P < 0.05; **P < 0.01; ^#^ *P* < 0.005; ^##^ *P* < 0.001 compared with control.

We next examined whether 4‐1BBL signaling in microglia enhanced inflammatory responses. Obesity‐related peripheral factors (LPS, FFA, high glucose, and obese ATCM) slightly upregulated the transcript levels of 4‐1BBL in microglia (BV2) (data not shown). To stimulate 4‐1BBL signaling in microglia, BV2 microglial cells were treated with r4‐1BB‐Fc, and the production levels of inflammatory cytokines were measured. 4‐1BBL stimulation on BV2 cells increased the transcripts levels of microglia activation markers such as Nos2, CD11b, and Iba‐1 (Fig. 3A). This was accompanied by activation of inflammatory signaling molecules such as phosphorylation of ERK and JNK (Fig. 3B). Consistent with this, 4‐1BBL stimulation on BV2 microglia markedly increased the transcripts of pro‐inflammatory cytokines (MCP‐1, IL‐6) (Fig. 3C). 4‐1BBL stimulation also increased the production of MCP‐1 and IL‐6 proteins from BV2 microglia (Fig. 3D).

### Increased production of inflammatory cytokines in cocultured astrocytes/microglia

**Figure 4:**
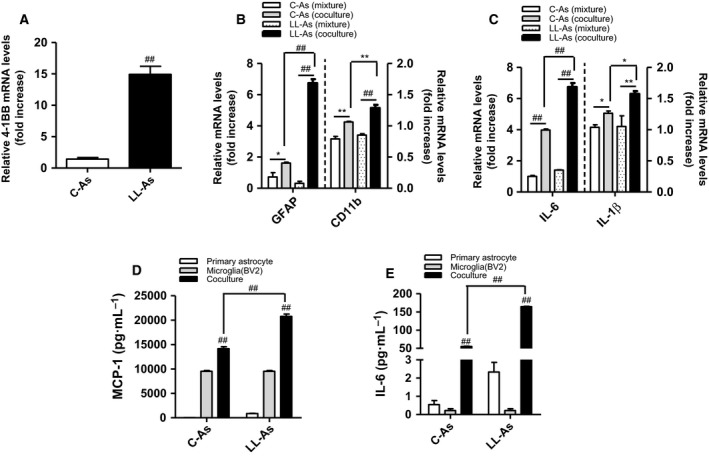
Inflammatory responses in cocultured astrocytes/microglia. Astrocytes were treated with 200 μm palmitic acid for 48 h. Microglia were directly seeded into the plates containing astrocytes. (A–C) The transcription levels of 4‐1BB, astrogliosis marker (GFAP), microglia activation marker (CD11b), and inflammatory cytokines (IL‐6, IL‐1β) were measured by qRT‐PCR. (D–E) MCP‐1, IL‐6 proteins were measured by ELISA. Data presented are representative of three independent experiments performed in triplicate. *P < 0.05; **P < 0.01; ^##^ *P* < 0.001 compared with control.

Using a contact coculture of astrocytes and microglia, we further examined whether 4‐1BB/4‐1BBL signaling mediates glial cell interactions, thereby enhancing the inflammatory responses. We first observed that 4‐1BB transcript was upregulated in lipid‐laden astrocytes, which contain lipid droplets in the palmitate‐rich obese condition (Fig. 4A). Subsequently, we found that direct contact coculture of astrocytes and microglia markedly increased the transcription of astrogliosis marker (GFAP) and microglia activation marker (CD11b) (Fig. 4B), and the coculture also increased the transcript levels of inflammatory cytokines (IL‐6, IL‐1β) (Fig. 4C) as well as the release of MCP‐1 and IL‐6 (Fig. 4D,E). More importantly, we found that the cocultured lipid‐laden astrocytes/microglia released greater amounts of inflammatory cytokines (MCP‐1, IL‐6) than control cocultures of astrocytes (C‐As) and microglia (Fig. 4D,E).

### Disruption of 4‐1BB and 4‐1BBL interaction in cocultured lipid‐laden astrocytes/microglia

**Figure 5:**
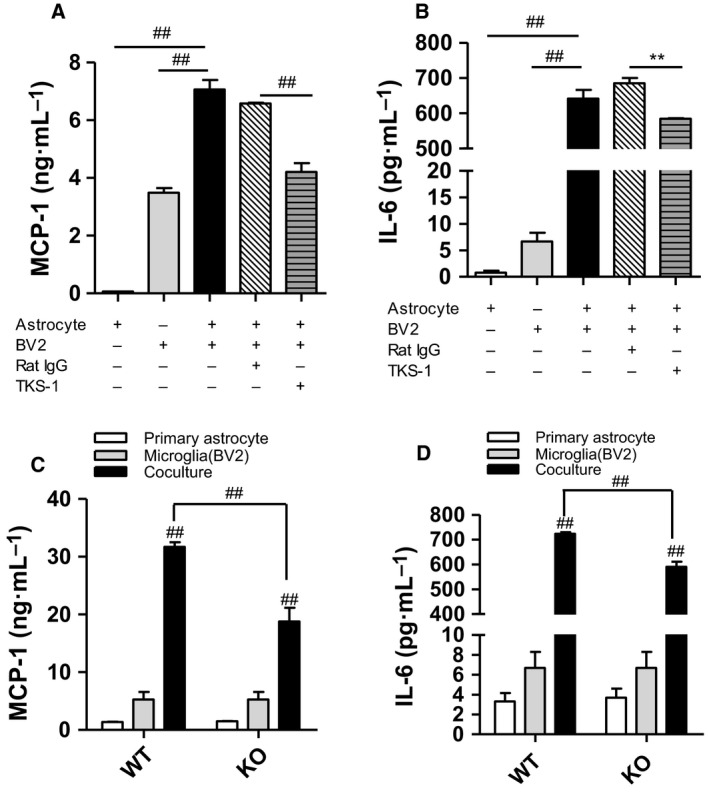
Effect of neutralizing antibody on 4‐1BB/4‐1BBL‐mediated inflammatory response in a contact coculture system. (A, B) Astrocytes were treated with 200 μm palmitic acid for 48 h. Microglia were seeded on to lipid‐laden astrocytes with or without pretreatment with neutralizing anti‐4‐1BBL antibody (TKS‐1) or rat IgG (5 μg·mL^−1^) in serum‐free medium for 24 h. (C, D) Astrocytes from WT or 4‐1BB KO were treated with 200 μm palmitic acid for 48 h. Microglia were directly seeded in to plates containing astrocyte. MCP‐1, IL‐6 proteins were measured by ELISA. Data presented are representative of three independent experiments performed in triplicate. **P < 0.01; ^##^ *P* < 0.001 compared with control.

We blocked cell–cell interactions using a neutralizing antibody (TKS‐1) in contact cocultured astrocytes/microglia. The neutralizing monoclonal antibody interrupts the interaction between 4‐1BBL and 4‐1BB by reacting specifically with mouse 4‐1BBL, and thus, 4‐1BBL cannot bind to the 4‐1BB receptor. Hence, both 4‐1BB‐mediated signaling in astrocytes and 4‐1BBL‐mediated signaling in microglia can be blunted by TKS‐1 treatment. We found that treatment with TKS‐1 significantly reduced releases of MCP‐1 and IL‐6 in the contact coculture microglia/astrocyte (Fig. 5A,B). Furthermore, using lipid‐laden astrocytes from 4‐1BB‐deficient mice or WT controls, we further examined whether 4‐1BB reduces the inflammatory responses of the glial cells in the cocultured condition. The production of inflammatory cytokines such as MCP‐1 and IL‐6 was markedly reduced in the cocultured 4‐1BB‐deficient astrocytes and microglia compared with the cocultured WT astrocytes and microglia (Fig. 5C,D). Given that blockade of the 4‐1BB/4‐1BBL interaction did not completely reduced the inflammatory responses of the cocultured glial cells, there may be other surface receptors/ligands involved in the glial cell‐mediated inflammatory responses.

**Figure 6:**
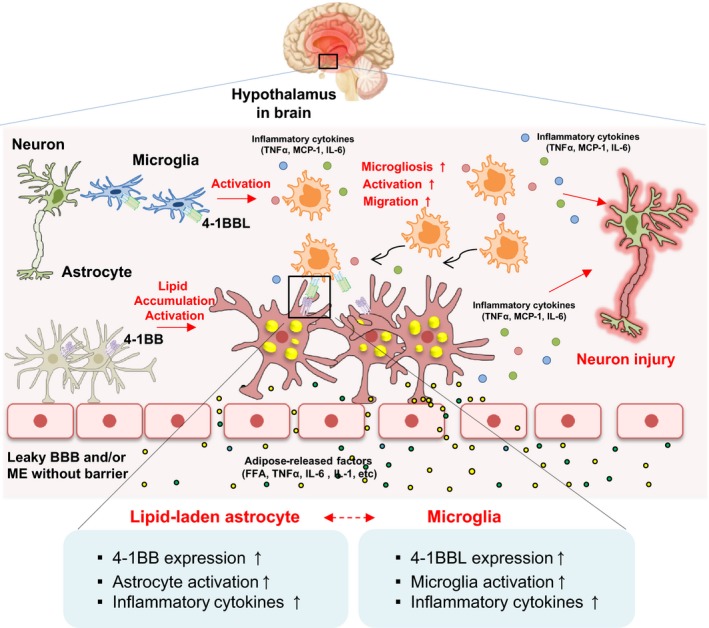
The involvement of 4‐1BB/4‐1BBL signaling in hypothalamic inflammation in obesity. 4‐1BB/4‐1BBL signaling enhances inflammatory reactivity of astrocytes and microglia to produce inflammatory mediators, and the glial cell‐mediated inflammatory cross talk is crucial for obesity‐induced hypothalamic inflammation.

## Discussion

Obesity/HFD‐induced hypothalamic inflammation is characterized by accumulation of glial cells (astrocytes and/or microglia) and their activation 11, 24, 25, 26, 27, leading to metabolic dysregulation including leptin/insulin resistance and thermogenic impairment 28, 29, 30. Several inflammatory mediators and their receptors have been shown to be expressed by glial cells and/or neurons, and their cross talk is implicated in central inflammatory pathologies. For example, several TNF receptor superfamily (TNFRSF)/ligands such as CD40/CD40L and TWEAK/FN14 also participate in glial cell‐mediated central/neuronal inflammatory diseases such as Alzheimer's disease and MS 31, 32 and are considered as a potential therapeutic target. However, whether glial cell–cell interaction mediates obesity‐induced hypothalamic inflammation and, if so, what molecules are involved in such cell–cell interactions, was not previously established. In this study, we showed for the first time that ablation of 4‐1BB, a member of TNFRSF, reduced transcript levels of inflammatory cytokines (TNFα, MCP‐1, and IL‐6) and gliosis markers (GFAP, Iba‐1, and CD11b) in hypothalamus of HFD‐fed obese mice, providing the first evidence that 4‐1BB signaling may participate in glial cell‐mediated hypothalamic inflammation in the HFD/obese condition.

Using primary astrocytes and microglia (BV2) *in vitro*, we further examined the direct involvement of 4‐1BB or 4‐1BBL in glial cell‐mediated inflammatory responses. Glial cells such as astrocytes and/or microglia increase their inflammatory reactivity when they accumulate lipid droplets under FFA‐rich condition like obesity 13, 33 and HFD‐fed mice 34, 35, and they release various mediators (cytokines/chemokines). We confirmed that the glial cells expressed 4‐1BB or 4‐1BBL mRNA, as reported previously 18, 20. Of note, we observed that transcript levels of 4‐1BB in astrocytes and 4‐1BBL in microglia were upregulated by the treatment of obesity‐related factors (LPS, FFA, glucose, ATCM), mimicking the obese condition. Consistent with this, we found that transcript levels of 4‐1BB and 4‐1BBL are upregulated in the hypothalamus of HFD‐fed obese mice compared with lean controls, indicating that 4‐1BB/4‐1BBL signals may enhance the inflammatory responses in glial cells under obese conditions. It was shown that activation of astrocytes accompanied by increased secretion of inflammatory cytokines is mediated through the NF‐κB pathway, and astrogliosis occurs through the activation of STAT3 36. Indeed, we observed that 4‐1BB stimulation induced degradation of the IκB‐α protein and activated STAT3, indicating that 4‐1BB signaling delivers an inflammatory signal to increase astrocyte reactivity. In line with this, we found that stimulation of 4‐1BB with an agonistic antibody on astrocytes markedly increased inflammatory cytokines TNFα, MCP‐1, and IL‐6 at transcript/protein levels, indicating that 4‐1BB signal promotes the astrocyte‐mediated inflammatory response.

Interestingly, 4‐1BBL also provides bidirectional signaling through binding to its ligand to 4‐1BB, which promotes monocyte/macrophage proliferation, migration, and inflammatory mediators 37. Hence, we hypothesized that 4‐1BBL signal has a role in microglia‐mediated inflammatory responses. Indeed, we found that 4‐1BBL stimulation in microglial cells increased microglia activation markers (Iba‐1, CD11b), and the expression and secretion of inflammatory mediators (MCP‐1, IL‐6), and reduced the expression of anti‐inflammatory cytokine IL‐10. A recent study showed that blocking the 4‐1BBL signal protects chemically induced neuronal injury, presumably by reducing microglial activation 38. Moreover, activation of the MAPK/NF‐κB pathway in microglia is known to induce neuroinflammation 27. In line with this, we observed that the 4‐1BBL‐stimulated inflammatory responses in microglia were mediated through activation of ERK and JNK protein, indicating that 4‐1BBL signal, which activates MAPK pathway, may be critical in microglia‐mediated inflammation.

We previously reported that lipid‐laden fatty astrocytes promote microglia migration and activation and thus may directly interact with the neighboring microglia, augmenting inflammatory responses 13. To obtain direct evidence of the involvement of 4‐1BB/4‐1BBL in the glial cell–cell interaction‐mediated inflammatory responses, we cocultured lipid‐laden astrocytes/macrophages. Indeed, the coculture of glial cells increased production of inflammatory cytokines and upregulated the activation markers in the cells. Notably, disruption of 4‐1BB/4‐1BBL interaction with a neutralizing antibody in the cocultured glial cells decreased the inflammatory responses of the cocultured lipid‐laden astrocytes/microglia. Accordingly, the reduction in the inflammatory responses was observed in 4‐1BB‐deficient astrocytes cocultured with microglia compared with those of WT astrocytes/microglia. These *in vitro* findings strongly support our *in vivo* observation that ablation of 4‐1BB reduces hypothalamic inflammation in HFD‐fed obese mice, which is accompanied by downregulation of glial cells activation markers and inflammatory cytokines levels. Taken together, these findings suggest that 4‐1BB and/or 4‐1BBL signaling has an important role in astrocytes/microglia‐mediated hypothalamic inflammatory responses under obese conditions.

In summary, we demonstrated for the first time that upregulation of 4‐1BB/4‐1BBL expression in the hypothalamus of HFD‐fed obese mice and ablation of 4‐1BB reduced hypothalamic inflammatory response in HFD‐fed obese mice. Stimulation of 4‐1BB or 4‐1BBL in lipid‐laden astrocytes or microglia increased the production of inflammatory mediators, and blockade of 4‐1BB/4‐1BBL interaction in cocultured glial cells with a neutralizing antibody reduced the inflammatory responses. These findings suggest that 4‐1BB/4‐1BBL signaling increases inflammatory reactivity of microglia and astrocytes to produce inflammatory mediators, leading to hypothalamic inflammation in obese condition (Fig. 6). 4‐1BB/4‐1BBL signaling may be a potential target for protection against obesity‐related hypothalamic inflammation and metabolic/neurodegenerative diseases.

## Author contributions

JK participated in design and coordination of the study, collected data and participated in data interpretations, and helped to draft the manuscript. Y‐HK participated in design and coordination of the study. C‐SK and THT researched data and contributed to discussion. B‐SK, YJ, HTC, TG, TK, TP, M‐SC, and M‐SK reviewed and approved the final manuscript. RY conceived of the study, participated in its design and coordination, participated in data interpretations, and drafted the manuscript. All authors read and approved the final manuscript.

## References

[1] Timper K and Bruning JC (2017) Hypothalamic circuits regulating appetite and energy homeostasis: pathways to obesity. Dis Model Mech 10, 679–689. 28592656 10.1242/dmm.026609 PMC5483000

[2] Thaler JP , Yi CX , Schur EA , Guyenet SJ , Hwang BH , Dietrich MO , Zhao X , Sarruf DA , Izgur V , Maravilla KR *et al* (2012) Obesity is associated with hypothalamic injury in rodents and humans. J Clin Invest 122, 153–162. 22201683 10.1172/JCI59660 PMC3248304

[3] Velloso LA , Araujo EP and de Souza CT (2008) Diet‐induced inflammation of the hypothalamus in obesity. Neuroimmunomodulation 15, 189–193. 18781083 10.1159/000153423

[4] Dwarkasing J , Marks D , Witkamp R and van Norren K (2016) Hypothalamic inflammation and food intake regulation during chronic illness. Peptides 77, 60–66. 26158772 10.1016/j.peptides.2015.06.011

[5] Lemus MB , Bayliss JA , Lockie SH , Santos VV , Reichenbach A , Stark R and Andrews ZB (2015) A stereological analysis of NPY, POMC, Orexin, GFAP astrocyte, and Iba1 microglia cell number and volume in diet‐induced obese male mice. Endocrinology 156, 1701–1713. 25742051 10.1210/en.2014-1961

[6] Garden GA and Moller T (2006) Microglia biology in health and disease. J Neuroimmune Pharmacol 1, 127–137. 18040779 10.1007/s11481-006-9015-5

[7] Iglesias J , Morales L and Barreto GE (2017) Metabolic and inflammatory adaptation of reactive astrocytes: role of PPARs. Mol Neurobiol 54, 2518–2538. 26984740 10.1007/s12035-016-9833-2

[8] Garcia‐Caceres C , Quarta C , Varela L , Gao Y , Gruber T , Legutko B , Jastroch M , Johansson P , Ninkovic J , Yi CX *et al* (2016) Astrocytic insulin signaling couples brain glucose uptake with nutrient availability. Cell 166, 867–880. 27518562 10.1016/j.cell.2016.07.028 PMC8961449

[9] Le Thuc O , Stobbe K , Cansell C , Nahon JL , Blondeau N and Rovere C (2017) Hypothalamic inflammation and energy balance disruptions: spotlight on chemokines. Front Endocrinol 8, 197. 10.3389/fendo.2017.00197 PMC5557773 28855891

[10] Milanski M , Degasperi G , Coope A , Morari J , Denis R , Cintra DE , Tsukumo DM , Anhe G , Amaral ME and Takahashi HK (2009) Saturated fatty acids produce an inflammatory response predominantly through the activation of TLR4 signaling in hypothalamus: implications for the pathogenesis of obesity. J Neurosci 29, 359–370. 19144836 10.1523/JNEUROSCI.2760-08.2009 PMC6664935

[11] Garcia‐Caceres C , Yi CX and Tschop MH (2013) Hypothalamic astrocytes in obesity. Endocrinol Metab Clin North Am 42, 57–66. 23391239 10.1016/j.ecl.2012.11.003

[12] Valdearcos M , Douglass JD , Robblee MM , Dorfman MD , Stifler DR , Bennett ML , Gerritse I , Fasnacht R , Barres BA , Thaler JP *et al* (2017) Microglial inflammatory signaling orchestrates the hypothalamic immune response to dietary excess and mediates obesity susceptibility. Cell Metab 26, 185–197.e3. 28683286 10.1016/j.cmet.2017.05.015 PMC5569901

[13] Kwon YH , Kim J , Kim CS , Tu TH , Kim MS , Suk K , Kim DH , Lee BJ , Choi HS , Park T *et al* (2017) Hypothalamic lipid‐laden astrocytes induce microglia migration and activation. FEBS Lett 591, 1742–1751. 28542876 10.1002/1873-3468.12691

[14] Drenkard D , Becke FM , Langstein J , Spruss T , Kunz‐Schughart LA , Tan TE , Lim YC and Schwarz H (2007) CD137 is expressed on blood vessel walls at sites of inflammation and enhances monocyte migratory activity. FASEB J 21, 456–463. 17167064 10.1096/fj.05-4739com

[15] Kim CS , Kim JG , Lee BJ , Choi MS , Choi HS , Kawada T , Lee KU and Yu R (2011) Deficiency for costimulatory receptor 4‐1BB protects against obesity‐induced inflammation and metabolic disorders. Diabetes 60, 3159–3168. 21998397 10.2337/db10-1805 PMC3219944

[16] Shao Z and Schwarz H (2011) CD137 ligand, a member of the tumor necrosis factor family, regulates immune responses via reverse signal transduction. J Leukoc Biol 89, 21–29. 20643812 10.1189/jlb.0510315

[17] Tu TH , Kim CS , Goto T , Kawada T , Kim BS and Yu R (2012) 4‐1BB/4‐1BBL interaction promotes obesity‐induced adipose inflammation by triggering bidirectional inflammatory signaling in adipocytes/macrophages. Mediators Inflamm 2012, 972629. 23316108 10.1155/2012/972629 PMC3534384

[18] Yeo YA , Martinez Gomez JM , Croxford JL , Gasser S , Ling EA and Schwarz H (2012) CD137 ligand activated microglia induces oligodendrocyte apoptosis via reactive oxygen species. J Neuroinflammation 9, 173. 22799524 10.1186/1742-2094-9-173 PMC3420242

[19] Le NH , Kim CS , Tu TH , Choi HS , Kim BS , Kawada T , Goto T , Park T , Park JH and Yu R (2013) Blockade of 4‐1BB and 4‐1BBL interaction reduces obesity‐induced skeletal muscle inflammation. Mediators Inflamm 2013, 865159. 24453430 10.1155/2013/865159 PMC3880756

[20] Reali C , Curto M , Sogos V , Scintu F , Pauly S , Schwarz H and Gremo F (2003) Expression of CD137 and its ligand in human neurons, astrocytes, and microglia: modulation by FGF‐2. J Neurosci Res 74, 67–73. 13130507 10.1002/jnr.10727

[21] Kwon BS , Hurtado JC , Lee ZH , Kwack KB , Seo SK , Choi BK , Koller BH , Wolisi G , Broxmeyer HE and Vinay DS (2002) Immune responses in 4‐1BB (CD137)‐deficient mice. J Immunol 168, 5483–5490. 12023342 10.4049/jimmunol.168.11.5483

[22] Shuford WW , Klussman K , Tritchler DD , Loo DT , Chalupny J , Siadak AW , Brown TJ , Emswiler J , Raecho H , Larsen CP *et al* (1997) 4‐1BB costimulatory signals preferentially induce CD8+ T cell proliferation and lead to the amplification in vivo of cytotoxic T cell responses. J Exp Med 186, 47–55. 9206996 10.1084/jem.186.1.47 PMC2198949

[23] McCarthy KD and de Vellis J (1980) Preparation of separate astroglial and oligodendroglial cell cultures from rat cerebral tissue. J Cell Biol 85, 890–902. 6248568 10.1083/jcb.85.3.890 PMC2111442

[24] Tran DQ , Tse EK , Kim MH and Belsham DD (2016) Diet‐induced cellular neuroinflammation in the hypothalamus: mechanistic insights from investigation of neurons and microglia. Mol Cell Endocrinol 438, 18–26. 27208620 10.1016/j.mce.2016.05.015

[25] Lively S and Schlichter LC (2013) The microglial activation state regulates migration and roles of matrix‐dissolving enzymes for invasion. J Neuroinflammation 10, 75. 23786632 10.1186/1742-2094-10-75 PMC3693964

[26] Gao Y , Ottaway N , Schriever SC , Legutko B , García‐Cáceres C , Mergen C , Bour S , Thaler JP , Seeley RJ and Filosa J (2014) Hormones and diet, but not body weight, control hypothalamic microglial activity. Glia 62, 17–25. 24166765 10.1002/glia.22580 PMC4213950

[27] Dheen ST , Kaur C and Ling EA (2007) Microglial activation and its implications in the brain diseases. Curr Med Chem 14, 1189–1197. 17504139 10.2174/092986707780597961

[28] Romanatto T , Cesquini M , Amaral ME , Roman EA , Moraes JC , Torsoni MA , Cruz‐Neto AP and Velloso LA (2007) TNF‐alpha acts in the hypothalamus inhibiting food intake and increasing the respiratory quotient–effects on leptin and insulin signaling pathways. Peptides 28, 1050–1058. 17459524 10.1016/j.peptides.2007.03.006

[29] Romanatto T , Roman EA , Arruda AP , Denis RG , Solon C , Milanski M , Moraes JC , Bonfleur ML , Degasperi GR , Picardi PK *et al* (2009) Deletion of tumor necrosis factor‐alpha receptor 1 (TNFR1) protects against diet‐induced obesity by means of increased thermogenesis. J Biol Chem 284, 36213–36222. 19858212 10.1074/jbc.M109.030874 PMC2794737

[30] Morrison SF (2016) Central neural control of thermoregulation and brown adipose tissue. Auton Neurosci 196, 14–24. 26924538 10.1016/j.autneu.2016.02.010 PMC4846468

[31] Benveniste EN , Nguyen VT and O'Keefe GM (2001) Immunological aspects of microglia: relevance to Alzheimer's disease. Neurochem Int 39, 381–391. 11578773 10.1016/s0197-0186(01)00045-6

[32] Giunta B , Rezai‐Zadeh K and Tan J (2010) Impact of the CD40‐CD40L dyad in Alzheimer's disease. CNS Neurol Disord Drug Targets 9, 149–155. 20205645 10.2174/187152710791012099 PMC2892111

[33] Yang J , Kim CS , Tu TH , Kim MS , Goto T , Kawada T , Choi MS , Park T , Sung MK , Yun JW *et al* (2017) Quercetin protects obesity‐induced hypothalamic inflammation by reducing microglia‐mediated inflammatory responses via HO‐1 induction. Nutrients, 9, 650. 10.3390/nu9070650 PMC5537770 28644409

[34] Lee JJ , Wang PW , Yang IH , Huang HM , Chang CS , Wu CL and Chuang JH (2015) High‐fat diet induces toll‐like receptor 4‐dependent macrophage/microglial cell activation and retinal impairment. Invest Ophthalmol Vis Sci 56, 3041–3050. 26024088 10.1167/iovs.15-16504

[35] Douglass J , Dorfman M , Fasnacht R , Shaffer L and Thaler J (2017) Astrocyte IKKβ/NF‐κB signaling is required for diet‐induced obesity and hypothalamic inflammation. Mol Metab 6, 366–373. 28377875 10.1016/j.molmet.2017.01.010 PMC5369266

[36] Ben Haim L , Carrillo‐de Sauvage MA , Ceyzeriat K and Escartin C (2015) Elusive roles for reactive astrocytes in neurodegenerative diseases. Front Cell Neurosci 9, 278. 26283915 10.3389/fncel.2015.00278 PMC4522610

[37] Langstein J , Michel J , Fritsche J , Kreutz M , Andreesen R and Schwarz H (1998) CD137 (ILA/4‐1BB), a member of the TNF receptor family, induces monocyte activation via bidirectional signaling. J Immunol 160, 2488–2494. 9498794

[38] Malon J and Cao L (2015) Involvement of CD137L in peripheral nerve injury‐induced neuropathic pain behaviors (CAM1P.161). J Immunol 194, 48.18.

